# Green synthesis and characterization of Ag nanoparticles from *Mangifera indica* leaves for dental restoration and antibacterial applications

**DOI:** 10.1007/s40204-017-0067-9

**Published:** 2017-05-03

**Authors:** Dola Sundeep, T. Vijaya Kumar, P. S. Subba Rao, R. V. S. S. N. Ravikumar, A. Gopala Krishna

**Affiliations:** 1 School of Nanotechnology, Center for Nano Science and Technology, Institute of Science and Technology (IST), Jawaharlal Nehru Technological University, Kakinada, Andhra Pradesh 533 003 India; 2 Department of Physics, University College of Sciences, Acharya Nagarjuna University, Guntur, Andhra Pradesh India; 3 Department of Mechanical Engineering, K L University, Green Fields, Vaddeswaram, Guntur, Andhra Pradesh 522 520 India

**Keywords:** Silver nanoparticles, Ionomer cement, Vickers hardness, Monsanto hardness, Antibacterial activity

## Abstract

Green synthesis has gained a wide recognition as clean synthesis technique in the recent years. In the present investigation, silver nanoparticles were prepared by a novel green synthesis technique using *Mangifera indica* (Mango leaves) and found to be successfully used in dental applications. The prepared samples were spectroscopically characterized by XRD, PSA, SEM with EDS, and UV–Vis spectroscopy. The crystalline size and lattice strain were analyzed from the XRD data which were counter-verified by W–H plots and particle size analyzer. The XRD peaks revealed that average crystalline size of the as-synthesized Ag nanoparticles was of 32.4 nm with face-centered cubic structure. This was counter-verified by particle size analyzer and Williamson–Hall plots and found to be 31.7 and 33.21 nm in the former and latter, and the crystalline size of Ag NPs could be concluded as 32 ± 2 nm. The morphological structure of the prepared sample was studied through SEM images and the chemical composition was analyzed by the EDS data. The band energy was calculated as 393 nm from UV–Vis, which confirmed the synthesized sample as Ag nanoparticles. To improve the mechanical bonding and hardness of the dentally used glass ionomer cement (GIC), the synthesized silver nanoparticles were incorporated into GIC in 2% weight ratio. The morphology of the prepared specimens was studied using optical microscope images. Vickers microhardness and Monsanto hardness tests were performed on GIC, GIC reinforced with microsilver particles and GIC reinforced with nanosilver particles and the latter derived a promising results. The results of the Monsanto tests confirmed the increase in hardness of the GIC reinforced with AgNps as 14.2 kg/cm^2^ compared to conventional GIC and GIC reinforced with silver microparticle as 11.7 and 9.5 kg/cm^2^. Similarly the Vickers hardness results exhibited the enhanced hardness of GIC-reinforced AgNps as 82 VHN compared to GIC as 54 and GIC-reinforced silver microparticles as 61 VHN. The antibacterial activity of AgNPs was tested by a well-diffusion method on *Escherichia coli* and *Staphylococcus aureus* bacteria, and the obtained results exhibited a promising antibacterial activity of the as-synthesized nanoparticles.

## Introduction

The fascinating size of 1–100 nm is dealt with nanotechnology which has gained a wider interest in the research field. Nanotechnology has become a useful tool in medical field for its extensive applications in several forms. Metal nanoparticles are always enhanced for their unique properties and potential applications in the engineering and medical field. Among the various metal nanoparticles noble metal nanoparticles such as silver nanoparticles (AgNPs) were of great importance due to their distinctive physical and chemical properties. Recently the study of AgNPs is concentrated due to their antibacterial activity (Kim et al. 2007; Shahverdi et al. 2007) and dental applications (García-Contreras et al. 2011). AgNps are also used in various applications such as nanosensors due to their excellent electrochemical properties (Manno et al. 2008), halloysite nanotubes (HNTs) (Liu and Zhao 2009), catalyst (Guo et al. 2008), textile industry, water treatment (Dankovich and Gray 2011) optical data storage (Kelly et al. 2003), fluorescent emissions in biological labels and electroluminescent displays (Mulvaney 1996; Berciaud et al. 2005).

Glass ionomers (GI) were an important adhesion of restorative materials in restorative dentistry. The glass ionomer cement (GIC) is composed of acid and base, i.e., calcium, strontium aluminosilicate glass powder (base) combined with a water-soluble polymer (acid) such as aqueous solution of an acrylic acid homo- or copolymer. This glass ionomer cement finds a wider area of applications due to its unique properties and is used as base metals in restorative and adhesive to tooth structure. They are also used for its anticarcinogenic, thermal compatibility and biocompatibility properties. The invention of glass ionomer cement replaced the conventional cements made of phosphoric and silicate in dental medicine. GIC finds numerous applications in dentistry medicine such as to treat luting crowns, bridges, esthetic restorative cement, reinforced restorative cement and lining cement. They provide tooth-colored restorations with low technique sensitivity and bond chemically to fix the decayed tooth and release levels of fluoride that protect cavosurface margins from recurrent caries attack (Knight 2016). The main disadvantages of the GICs are it is not recommended for biting surfaces in permanent teeth, the aging of the GIC results in poor wear and cracks over time and also accumulates plaque and periodontics diseases due to accumulation of bacteria commonly known as secondary caries.

Secondary caries are the most vulnerable caries raised out after different dental restoration treatments and the most commonly raised after fixing the caries or tooth decays with GIC. The placement of restorations can lead to the development of environmental conditions favorable to microbial colonization, especially on the tooth/restoration interface, which is a predisposing factor for secondary caries (Pedrini et al. 2001).

In the present work, the AgNps were synthesized using a novel green synthesis technique (Kouvaris et al. 2012). Various other synthesis techniques have also been reported for the preparation of metal silver nanoparticles such as laser ablation (Bae et al. 2002), microwave–polyol (Patel et al. 2005a, b), sonochemical (Salkar et al. 1999; Zhang et al. 2006), sonoelectrochemical (Liua et al. 2001), microwave dielectric heating (Patel et al. 2005a, b) solvothermal (Rosemary and Pradeep 2003) and electrochemical (Starowicz et al. 2006) synthesis techniques. Most of these conventional physical and chemical nanoparticle synthesis techniques results in the usage of toxic chemicals and are expensive. Efforts are being made to develop environmentally friendly synthesis techniques to fabricate nanoparticles (Hubenthal (2011). Some of the most important fabrication techniques of green synthesis are achieved through bio-organisms, plant leaves and fruit extracts (Ghodake et al. 2010). Cost efficiency, fast and low risks of toxicity are the important advantages of green synthesis (Sanghi and Verma 2009).

To the best of our knowledge there are no reports on the green-synthesized Ag nanoparticles from *Magnifera Indica* leaves which are used for dual, i.e., antimicrobial and dental restoration application at a single instance. In the present work, AgNPs were prepared by a novel green synthesis technique. Sumit Kumar et al., Martínez-Bernett et al. and Vikas Sarsar et al. used the green synthesis technique to produce the silver nanoparticles. Sumit Kumar et al. worked on the synthesis of silver nanoparticle using a combination of *Magnifiera indica* and *Syzygium cumini* leaf extracts. Martínez-Bernett et al. and Vikas Sarsar et al. also presented the synthesis of silver nanoparticles from *Magnifiera indica* leaf extracts, but they did not project the antimicrobial activity or the usage of dental restoration applications using the prepared silver nanoparticles (Contreras et al. 2015; Martínez-Bernett et al. 2016; Kumar et al. 2013, Sarsar et al. 2013; Philip 2011). In the present work, we elevated the combinational, i.e., dual application of the silver nanoparticles as an antimicrobial agent and even for the dental restoration. Their structural properties are reported by spectroscopic characterization such as Particle Size Analyzer (PSA), Powder X-ray diffraction (PXRD), Scanning Electron Microscope (SEM), Energy-dispersive X-ray analysis (EDS) and UV–Vis spectroscopy. The as-synthesized AgNPs were reinforced with glass ionomer cement to improve the mechanical bonding strength. The Stokes Monsanto hardness and Micro Vickers Hardness tests were performed on GIC, GIC reinforced with microsilver particles and GIC reinforced with AgNPs. The antimicrobial activity of the prepared AgNPs was tested on *E. coli* and *S. aureus* bacterial species.

The eventual rational of the present work is to synthesis a clean and eco-friendly, economic and tranquil bulk silver nanoparticle. These prepared silver nanoparticles were used in a two-way dental restoration application simultaneously. As previously mentioned, the dentistry medication with glass ionomer cement finds a major limitation in poor wear and secondary caries raised out of accumulation of bacterial colonies around the restoration using GIC at an early or aging state. The prepared silver nanoparticles were reinforced with glass ionomer cement to meet the said two limitations simultaneously. The reinforcement of AgNPs in GIC provides the enhancement in the hardness of conventional GIC and similarly resolves the limitation secondary caries raised of bacterial colonies around the GIC fixed restoration in post-medication. The enhancement of hardness of AgNP-reinforced GIC is compared with conventional GIC and microsilver-reinforced GIC and the antimicrobial activity of AgNPs is performed on Escherichia coli and Staphylococus aureus bacteria.

## Experimental procedure

Silver nanoparticles were synthesized by a novel green synthesis technique (Knight 2016). Fresh *Mangifera indica* (Mango leaves) were collected from the mango garden at Jawaharlal Nehru Technological University Kakinada, India and silver nitrate (AgNO_3_) was purchased from Merck chemicals, India. All the chemicals used in the production were of analytical grade and are of above 99% purity. These fresh mango leaves were washed thoroughly with distilled water, chopped into small pieces and air dried, respectively. About 15 gm of air dried leaf was weighed on a digital balance and mixed thoroughly with 60 ml of distilled water in a 250-ml beaker and boiled at 100 °C for 25 min. The extract obtained was cooled and filtered through Whatman No. 1 paper and the filtrate was collected in a 250-ml Erlenmeyer flask and stored in refrigerator for further use. In the typical synthesis of AgNPs, the leaf extract of 1.5 ml was added to 30 ml of 10^−3^ M AgNO_3_ solution in a 250-ml Erlenmeyer flask and heated on water bath at 75 °C for 1 h. Reduction of silver nitrate to silver ions was confirmed by the change in color of the prepared colorless solution to brown solution. The fully reduced solution was centrifuged at 2000 rpm for 30 min. The supernatant liquid was discarded and the viscous silver remnant was collected and applied over a Whatman No. 1 paper and left for 48 h. Now the dried silver nanoparticles were collected. Hence, AgNps were successfully synthesized.

### Hardness test specimens

The glass ionomer cement of type 9 (GIC 9) is blended with 3% weight ratio (Contreras et al. 2015) of the prepared face-centered cubic AgNPs and microsilver particles separately in a vortex for 2 min. Three different compounds were prepared in Teflon medium as GIC, GIC (3.4gms) reinforced 3% (w/w) (0.1 g) microsilver particles and GIC (3.4 g) reinforced 3% (w/w) (0.1 g) AgNPs. According to the American Dental Association (ADA) specification, 27 of these samples were prepared as 9.5 × 1 mm cylinder specimens Contreras et al. 2015) to undergo the Monsanto hardness and Vickers microhardness tests.

### Antibacterial assays

**Fig. 1:**
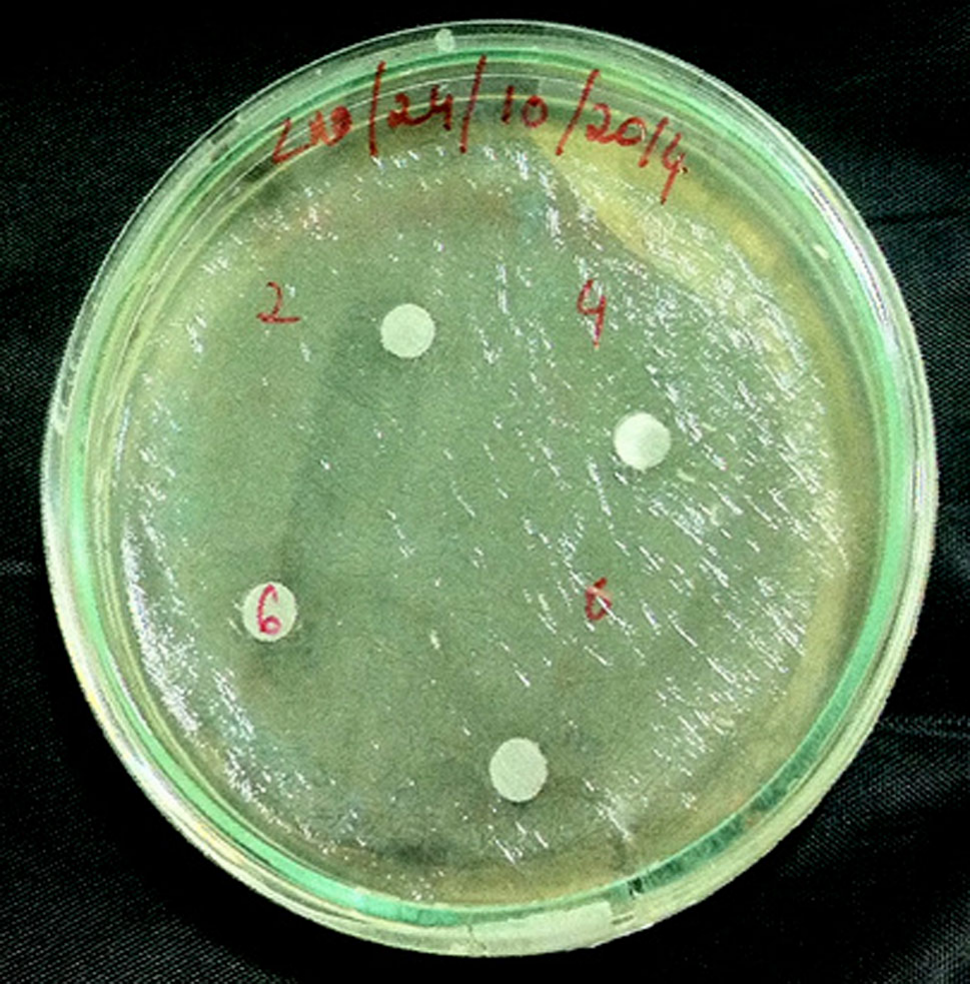
*Escherichia coli* Petri plates with Ag nanoparticles at different concentrations

The antibacterial effect of AgNPs was evaluated by standard diffusion technique or Kirby–Bauer methods (Sanghi and Verma 2009). Kirby–Bauer and Stokes methods are generally used for antibacterial testing. The most accepted antimicrobial susceptibility testing by Clinical and Laboratory Standards Institute (CLSI) is Kirby–Bauer method (Martínez-Bernett et al. 2016). 500 µl of microbial cultures of age 18–24 h were inoculated into Petri plates in nutrient agar medium and solidified. AgNPs were suspended in the four different holes as shown in Fig. 1 in four different concentrations of 2, 4, 6 and 8 µg/ml. These plates were wrapped with a parafilm tape and incubated for 24 h at 35 °C.

## Characterization

The VASCO™ nanoparticle size analyzer (PSA) was performed for nanoparticle characterization, based on the Dynamic Light Scattering (DLS) at 135 °C. Powder X-ray diffraction (PXRD) analysis were performed on a PANalytical XPert Pro-diffractometer equipped with Ni filter with CuKα radiation (*λ* = 1.5406 Å). The measurements were made at room temperature at a range of 10°–80° on 2*θ* with a step size of 0.05° in 40 keV at 15 mV applied current. Scanning electron microscope (SEM) images were obtained from a Carl Zeiss SEM EVO with carbon coating and energy-dispersive X-ray spectroscopy (EDS) images were taken from ZEISS EVO 18. Prepared powder sample was mixed with Nujol (liquid paraffin) mull form to record the optical absorption spectrum, and recorded on JASCO V-670 Spectrophotometer in region of 200–1400 nm wavelength at room temperature. Optical microscopic images of ionomer cement along with reinforced micro- and nanosilver particles were taken on Olympus BX61 digital microscope. The hardness of these samples was analyzed by HVS-1000Z Digital Micro Vickers Hardness Tester with 25 gram of load weight and Dolphin Stokes Monsanto hardness tester.

## Results and discussion

### Particle size analysis

**Fig. 2:**
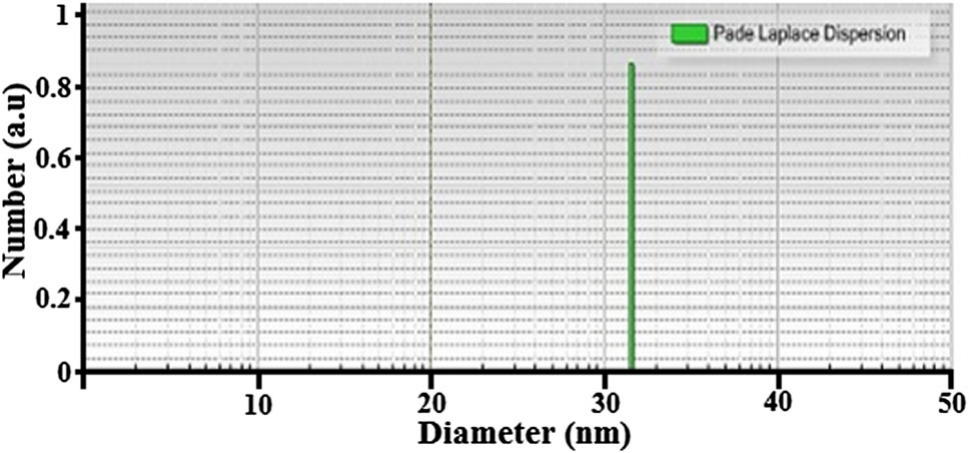
Particle size analyzer graph of Ag nanoparticles

The prepared AgNPs were suspended in low-concentrated ethyl alcohol and ultrasonicated for 10 min. The prepared sample was subjected 245 nm laser wavelength in particle size analyzer spectroscopy. Figure 2 shows the PSA graph of AgNPs. The synthesized AgNPs exhibit a mean particle size of 31.7 nm under the dynamic light scattering. The crystalline size calculated from PSA is further counter-verified by PXRD and Williamson–Hall plots.

### X-ray diffraction analysis

**Fig. 3:**
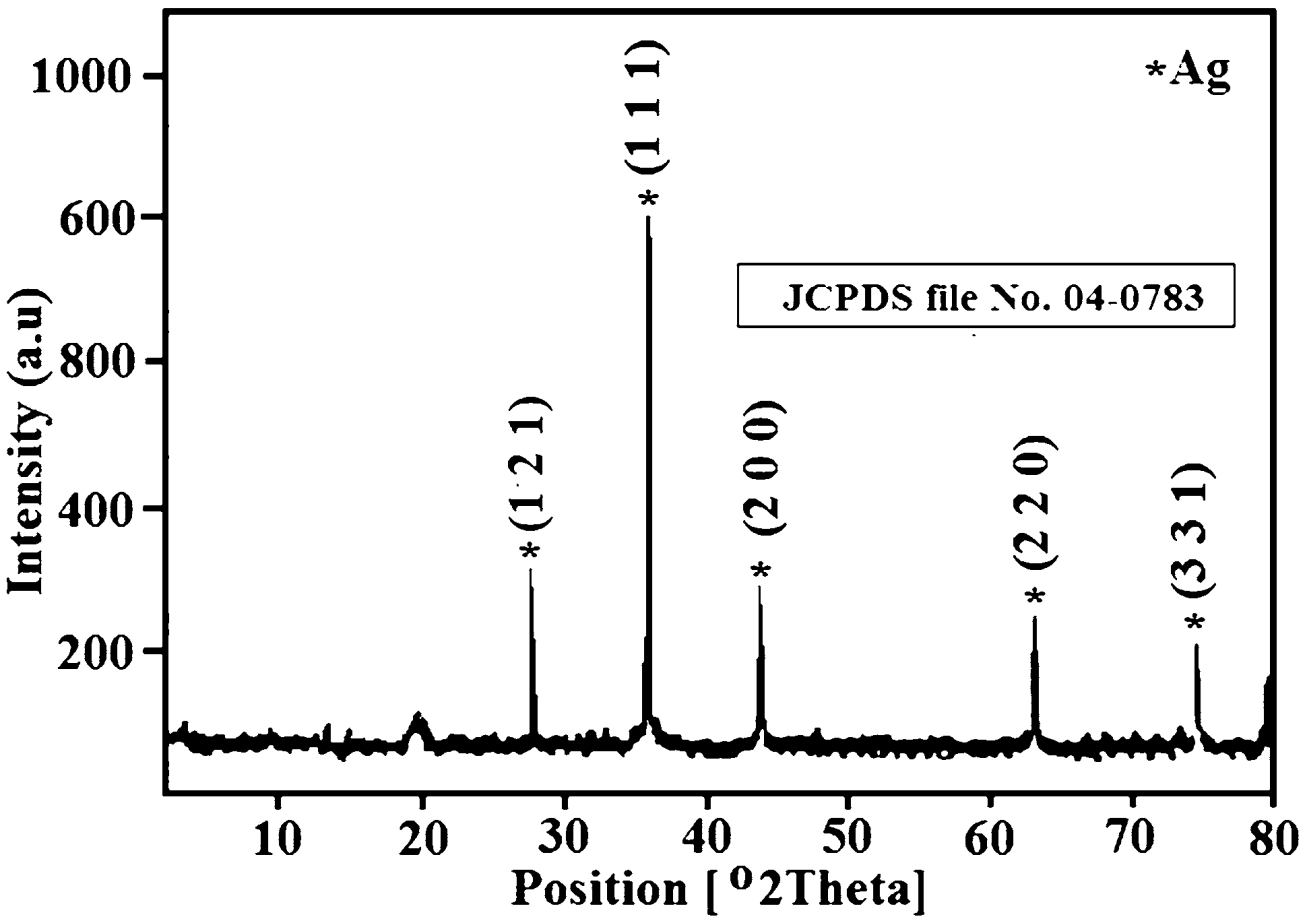
X-ray diffraction patterns of Ag nanoparticles

The X-ray diffraction is performed to estimate the crystal structural phase of the synthesized AgNPs. The XRD graph of AgNPs is as shown in Fig. 3. The diffraction peaks of AgNPs exhibit face-centered cubic (cF) crystal system with corresponding lattice parameters as *a* = 4.070 nm. All the diffraction peaks of the AgNPs are well matched with the standard diffraction data of JCPDS file No. 04-0783 for face-centered cubic Ag.

The existence of sharp peaks of 2*θ* located at 38.25°, 44.12°, 64.27° and 77.52° corresponding to (111), (200), (220) and (331) planes indicates the formation of pure silver nanoparticles. X-ray diffraction is a powerful tool used to estimate the crystalline size, lattice strain and dislocation densities. The average crystalline size of the prepared samples was calculated using the Debye–Scherrer’s formula given in the following equation (Sundeep et al. 2016; Gopala Krishna et al. 2016; Ravikumar et al. 2016)

1 $$ D = \frac{0.9\lambda }{\beta \,\cos \theta }\,\,{\text{nm}} $$

where ‘*D*’ is the crystalline size; *λ* is the wavelength of X-ray (*λ* = 0.154056 nm for (CuKα); *β* is the full width at half maximum (FWHM) of the Braggs peak (in radians); *θ* is the diffraction angle of the reflection.

From Eq. (1) the crystalline size is calculated as 32.3 nm, respectively. There is negligible increase in the crystalline size from XRD data in comparison to PSA. The sharp peaks assume the formation of pure sample with no evidence of any bulk remnants or impurities. Microstrain is a measurement of distribution of lattice constants arising from crystal imperfection such as lattice dislocation. The slight peak broadening in the XRD graph as shown in Fig. 3 indicates the presence of small microstrains in the sample. The microstrain (*ε*)-induced broadening in the powders are due to crystal imperfection and distortion which is calculated using the following equation (Gopala Krishna et al. 2016, Ravikumar et al. 2016).

2 $$ \varepsilon = \frac{\beta \cos \theta }{4} $$

X-ray line broadening was used to estimate the dislocation densities in the samples. The dislocation density (*δ*) was calculated using the following equation:

3 $$ \left( {\delta = 1/D^{2} } \right) $$

where *D* is the crystallite size.

Williamson–Hall analysis is a simplified integral breadth method employed for estimating crystal, crystallite size and lattice strain, considering the peak width as a function of 2*θ* (Sundeep et al. 2016). The crystallite size is not necessarily the same as particle size, since the crystallite size is assumed to be the size of a coherently diffracting domain. From Eqs. (1) and (2), it is clear that the peak width from crystalline size varies as per the equation $\frac{1}{\cos \theta }$ and strain takes the equation tan*θ*. Assuming the particle size and the strain contributions to line broadening are independent of each other and both have Cauchy-like profile, the observed line breadth is taken as the sum of Eqs. (1) and (2),

4 $$ \beta = \frac{0.9\lambda }{D\cos \theta } + 4\varepsilon \tan \theta $$

By rearranging Eq. (4), we get

5 $$ \beta \cos \theta = \frac{0.9\lambda }{D} + 4\varepsilon \sin \theta $$

**Fig. 4:**
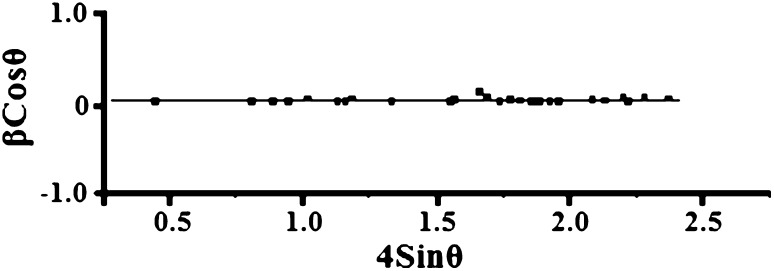
Williamson–Hall plot of Ag nanoparticles

Equation (5) is the Williamson–Hall equation (Sundeep et al. 2016), which assumes that the strain is uniform in all crystallographic directions. W–H equation represents a straight line between the 4sin*θ* (*X*-axis) and *β*cos*θ* (*Y*-axis). The crystallite size (*D*) and microstrain (*ε*) are calculated from the intercept (*kλ*/*D*) and slope of the line. Figure 4 shows the (W–H) plots of AgNPs. Table 1 shows the comparison of average crystalline size, lattice strain and dislocation density of the AgNPs. The crystalline sizes from particle size analyzer (PSA), Debye–Scherrer’s and microstrains calculated are compared with the calculations of W–H plots, and are found to be closely matched.

**Table 1:** Average crystallite size, lattice strain and dislocation densities of Ag nanoparticles

Sample	Crystalline size (nm)			Microstrain (*ε* × 10^−3^)		Dislocation density (*δ* × 10^−15^ m^−2^)
Silver nanoparticles	PSA	Scherrer’s	W–H method	Calculated	W–H method	Scherrer’s
	31.7	32.4	33.21	0.21	0.29	1.642

### Morphological studies

**Fig. 5:**
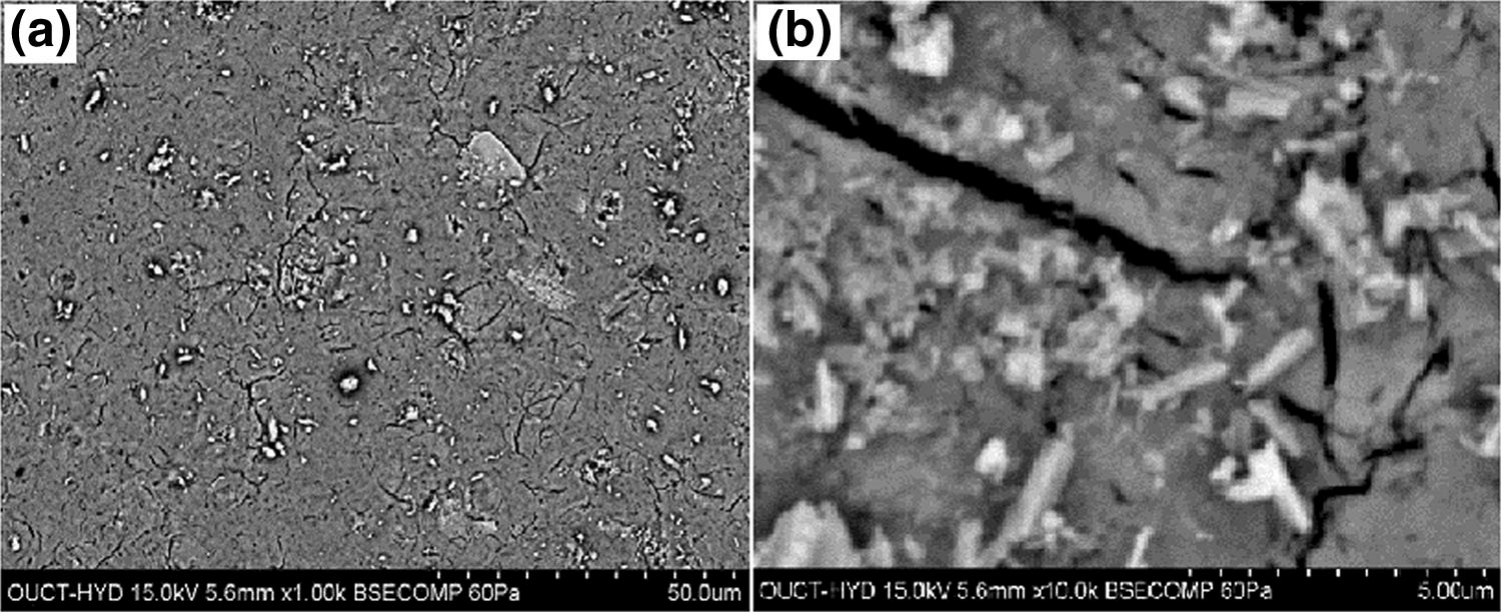
SEM images of Ag nanoparticles

**Fig. 6:**
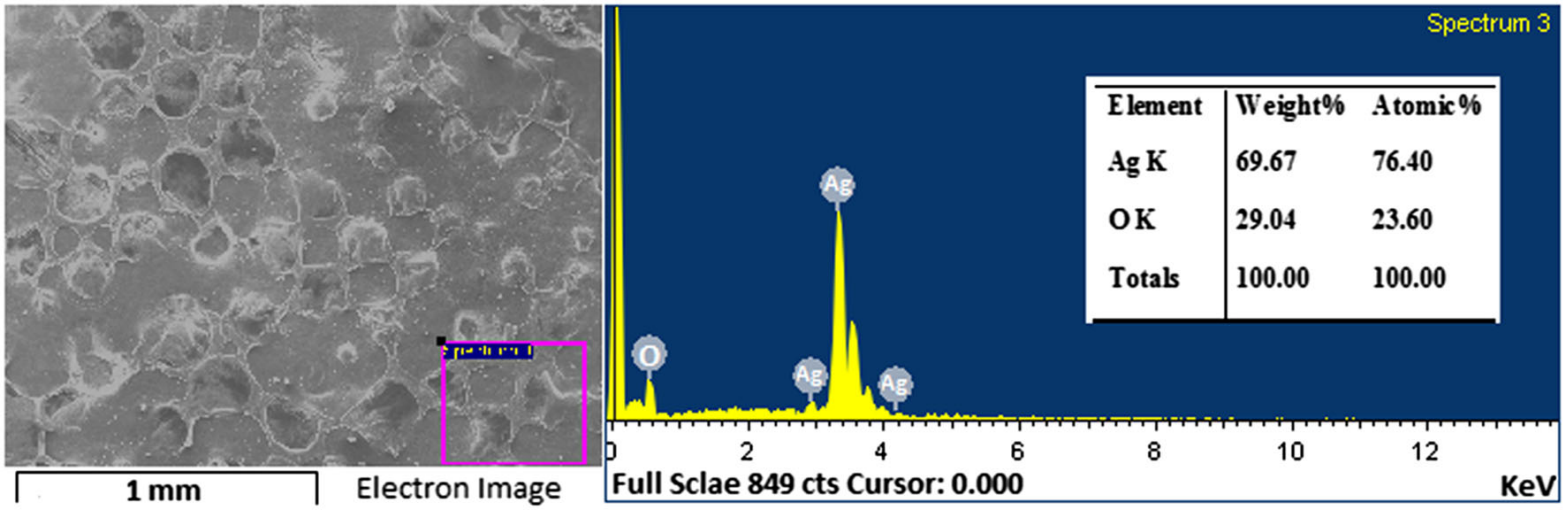
EDS pattern of Ag nanoparticles

The morphological studies of the prepared samples are observed by scanning electron microscopy analysis. To analyze the morphology, SEM is considered as an important technique (Sundeep et al. 2016). Figure 5 shows the SEM graphs of the Ag nanoparticles. SEM graphs reveal the flake-like structures of the synthesized AgNPs. The energy-dispersive X-ray spectra of AgNPs are shown in Fig. 6. The EDS spectroscopy is a potential tool used to reveal the elemental constituents. From the Fig. 6 it is clearly shown that the prepared sample is composed of Ag (silver) and O (oxygen). No other elemental impurity was found in EDS which strengths the purity of the sample.

### Optical studies

**Fig. 7:**
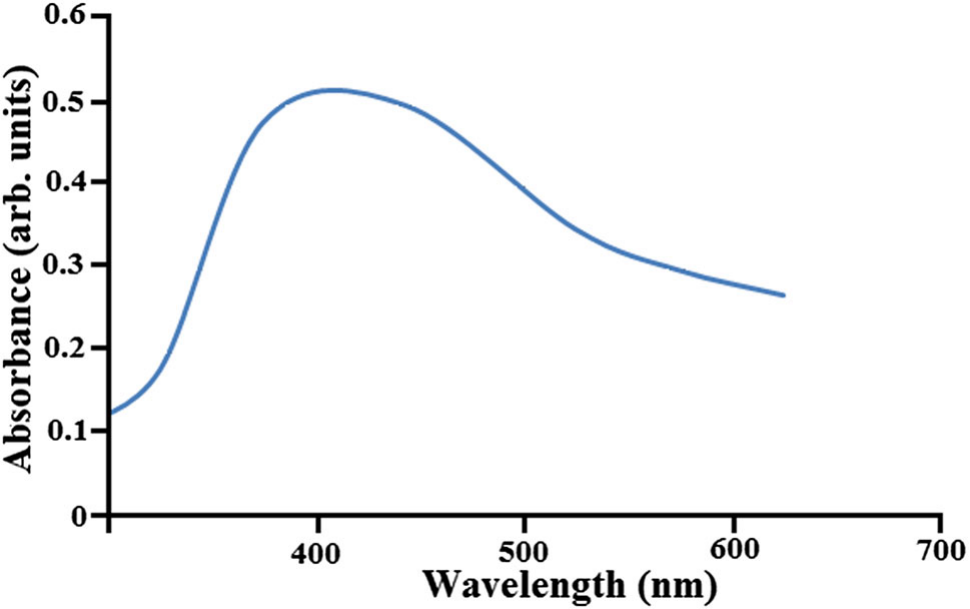
UV–Vis spectra of Ag nanoparticles

The optical properties of the synthesized AgNPs were analyzed by UV–Visible absorbance spectroscopy. The UV–Vis absorption spectrum of AgNPs is as shown in Fig. 7. Measuring the band gap is an important parameter in the nanomaterial industry. To analyze the conductivity of the synthesized nanomaterial, the term band gap refers to the energy difference between the top of the valency band and bottom of the conduction band which are able to jump from one band to another. For an electron to jump from a valence band to conduction band it requires a specific minimum amount of energy for the transition called as band energy. Based on the band gap energies the materials can be classified as insulators (>4 eV) and semiconductors (<3 eV). The optical edge of AgNPs nanoparticles is observed at 393 nm, which is a typical absorption band of face-centered cubic AgNPs (Alan Creighton et al. 1979). The band gap energy is calculated as 4.9 eV by the following Planck’s equation (Ma et al. 2015).

6 $$ {\text{Band Gap Energy }}\left( E \right) \, = \, h \, \times \, C/\lambda $$

where *h* is the Planck’s constant = 6.626 × 10^−34^ J s, *C* is the speed of light = 3 × 10^8^ m/s, and *λ* is the cutoff wavelength = 393 × 10^−9^.

## Dental and antimicrobial applications of AgNPs

In this part the green-synthesized AgNPs were reinforced with Glass ionomer cement (GIC) and the mechanical bonding enhancement was tested and compared with conventional and micro- and nanosilver particle-reinforced GIC. To analyze the antibacterial activity, AgNPs were tested on *E. coli* and *S. aureus* bacterial species.

### Dental application

Recently silver compounds and nanoparticles are studied for their vast area of research in dental applications such as dental restorative material, endodontic retro fill cement, dental implants and caries inhibitory solutions (Jia et al. 2008; Pissiotis and Spngberg 2000; Sheikh et al. 2010; Swift 1989). Glass ionomer cement (GIC) is known for its adhesiveness and biocompatibility; therefore, it is used as restorative material in dentistry (Pereira et al. 2002; Brentegani et al. 1997). Low wear resistance and facture toughness are the major disadvantages which result in the failure of restoration and leads to growth of bacterial proliferation. These failures can even lead to ancillary caries and tooth fracture. Extensive research had been done to overcome these failures and enhance the mechanical properties and bonding nature of GIC. Silver-amalgam particles, zirconia, spherical silica are some of the promising notable fillers reinforced with GIC to improve mechanical properties of GIC (Contreras et al. 2015).

**Fig. 8:**
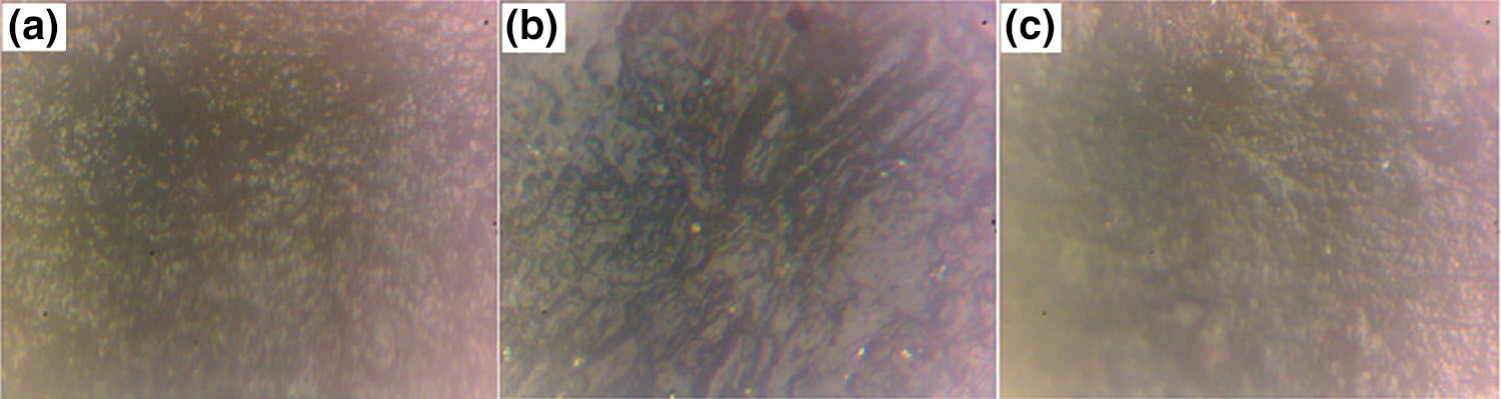
**a**–**c** Microscopic images of GIC, GIC-reinforced silver microparticles and GIC-reinforced silver nanoparticles

**Fig. 9:**
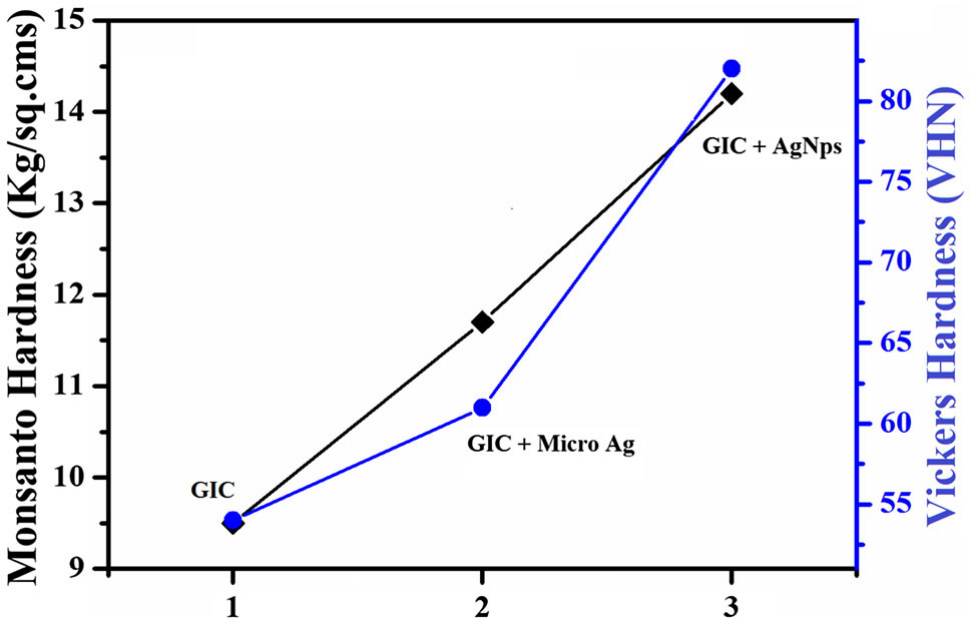
Graph comparing the average results of Monsanto and Vickers hardness tests

The microscopic images of GIC, GIC-reinforced microsilver particles and GIC-reinforced nanosilver particles are as shown in Fig. 8. The above-prepared compounds are divided into five samples and the mechanical hardness is tested by Monsanto and Vickers hardness tests. Hardness is the property of a material that enables it to resist plastic deformation, usually by penetration. Monsanto hardness tester consists of a barrel containing a compressible spring held between two plungers. The lower plunger is placed in contact with the tablet with a zero reading and the upper plunger is then forced against a spring by knurling the knob. The hardness is recorded in kg/sq.cms on breaking the pellet. The obtained Monsanto hardness results are tabulated in Table 2. Significant increase of mechanical bonding strength is achieved with AgNP-reinforced GIC. The Vickers microhardness test is the most sensitive test than Monsanto hardness test (Contreras et al. 2015). The above-prepared specimens were tested on diamond indenter with 25-g load weight. The results are tabulated in Table 2 and an improved mechanical bonding strength is observed with AgNP-reinforced specimens. The results were compared and are shown in Fig. 9.

**Table 2:** Monsanto and Vickers hardness values of GIC, GIC-reinforced microsilver particles and GIC reinforced with silver nanoparticles

S. no.	Hardness test					
	GIC		GIC reinforced with silver microparticles		GIC reinforced with silver nanoparticles	
	Monsanto (kg/cm^2^)	Vickers (VHN)	Monsanto (kg/cm^2^)	Vickers (VHN)	Monsanto (kg/cm^2^)	Vickers (VHN)
1	9.5	69	11.7	54	13.7	79
2	10.5	51	12.3	66	15.0	86
3	9.2	47	11.5	62	14.5	79
4	8.7	51	10.8	64	13.0	82
5	9.5	52	12.5	59	14.7	84
Average	9.5	54	11.7	61	14.2	82

### Antibacterial activity

**Fig. 10:**
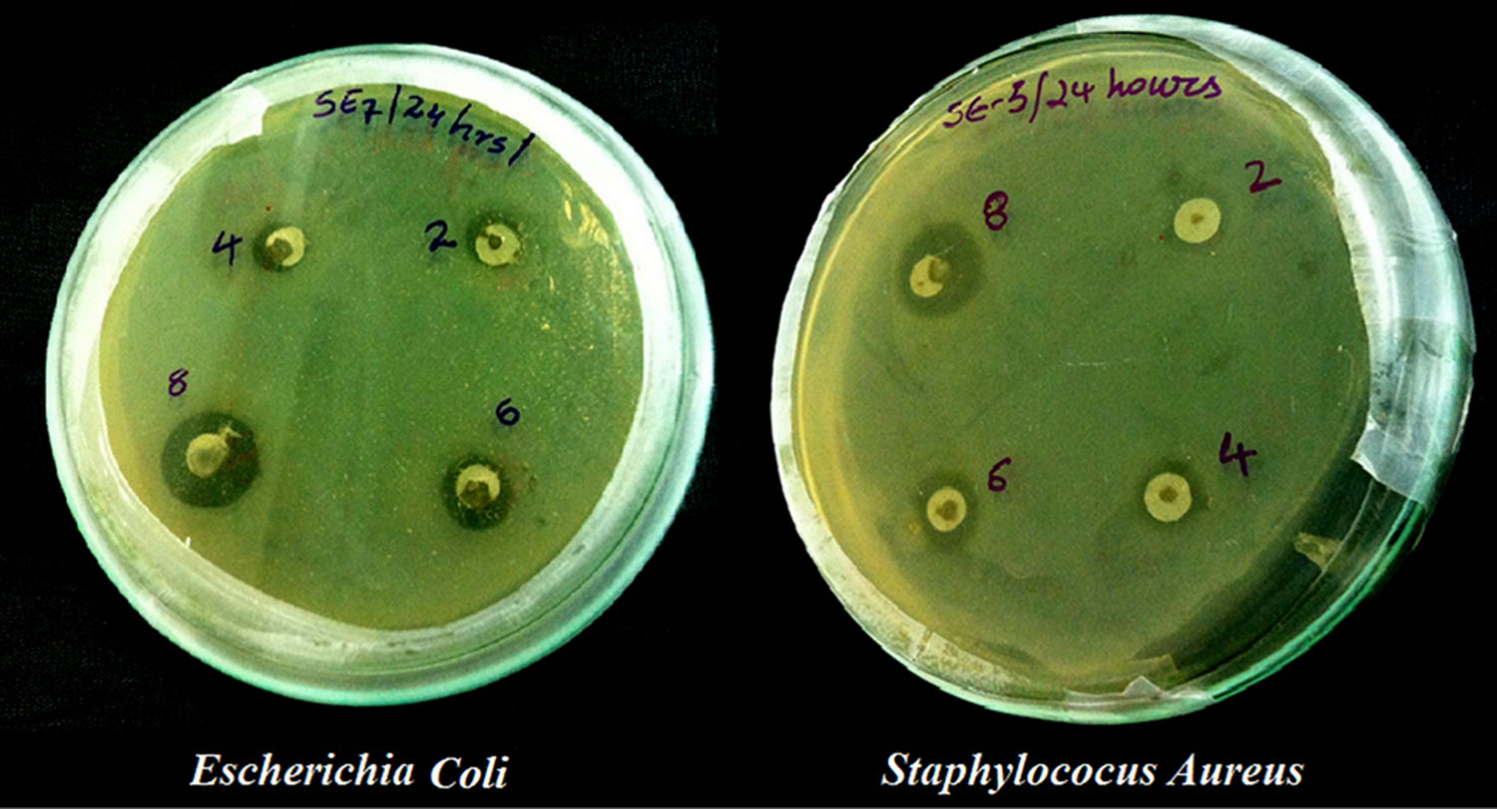
Antimicrobial activity of Ag nanoparticles at different concentrations against *Escherichia coli* and *Staphylococcus aureus*

**Fig. 11:**
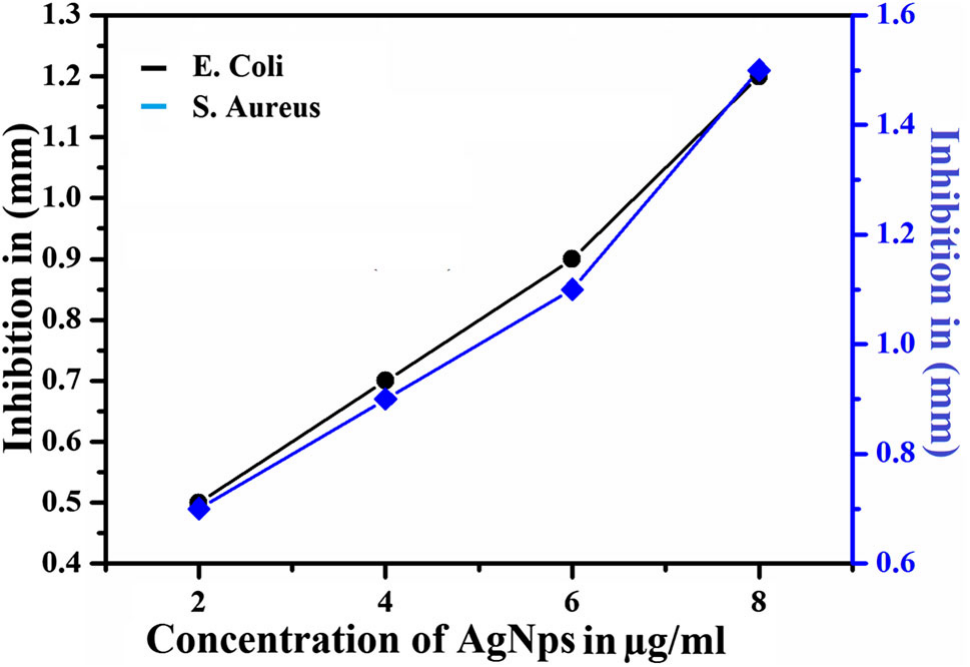
Zone of inhibition (mm) shown by AgNPs at different concentration

Silver ions show high biocidal effects on twelve different types of bacteria which include *E. coli* and *S. aureus* (Zhao and Stevens 1998). Even though the proper reasons for the antibacterial activity of AgNPs were not explained, Sondi, Salopek-Sondi et al. and Hamounda et al. explained the antibacterial effect of AgNPs as the electrostatic attraction between positively charged AgNPs and negatively charged bacteria (Sondi and Salopek-Sondi 2004; Sharma et al. 2009). Figure 10 shows the antibacterial effect of 2, 4, 6 and 8 µg/ml AgNPs on *E. coli* and *S. aureus* bacteria. More than 90% of the bacteria were inhibited by the presence of AgNPs. The inhibition zones were recorded in millimeters and are tabulated in Table 3 and are shown in Fig. 11. From Fig. 10 it is clearly shown that the suspended AgNPs inhibit the growth and reproduction of bacterial cells. It is clearly visible that after 24 h of inhibition almost all the bacterial cells were found dead and formed a layer around the AgNP pits in the Petri plates. The antibacterial activity of 2 µg/ml AgNPs was slightly slower than the other concentrations. From the concentration vs. inhibition graph (Fig. 11) the inhibition activity was ascending on increasing the concentration of AgNPs. Hence, this experiment can be considered for the usage of a controlled level of inhibition.

**Table 3:** Change of inhibition of *Escherichia coli* and *Staphylococcus aureus* with the concentration of AgNPs

Concentration of AgNPs	2 µg/ml	4 µg/ml	6 µg/ml	8 µg/ml
Inhibition zone of *Escherichia coli* (mm)	0.5	0.7	0.9	1.2
Inhibition zone of *Staphylococcus aureus* (mm)	0.7	0.9	1.1	1.5

## Conclusion

In this work, silver nanoparticles were synthesized by a novel green synthesis technique using fresh *Mangifera indica* (mango leaves) and characterized to evaluate the crystalline size, shape, crystalline imperfection and distortions, dislocation density. The crystalline size was evaluated from PXRD using the Scherrer’s formula and particle size analyzer. Both of the analysis evaluated the crystalline size as 32 ± 2 nm. The crystalline size was also evaluated theoretically using the Williamson–Hall plots and obtained the same crystalline size resulted from the PXRD and PSA analysis. The morphological studies were evaluated from the SEM images which confirm the nano-scale size of the synthesized particle. The EDS spectra showed the elemental composition of the prepared AgNPs and determined the purity of the sample. Optical studies were performed to evaluate the band gap energy and that was found to be 393 nm, which relates to the absorption band of face-centered cubic silver nanoparticles.

The Glass Ionomer Cement (GIC) was reinforced with as-synthesized AgNPs to obtain an enhanced dual dentistry application (improving the low wear of the conventional GIC and on the other hand, preventing the formation of bacterial colonies in the medicated part of the teeth). The Vickers microhardness test and Monsanto hardness test were performed on AgNP-reinforced GIC and the obtained results were compared with those of conventional GIC and GIC-reinforced microsilver particles. Results clearly showed the enhanced hardness of the AgNP-reinforced GIC. The antibacterial activity of the synthesized silver nanoparticles against *Escherichia coli and Staphylococcus aureus* bacteria was evaluated at different concentrations and reasonable antibacterial activity of the AgNPs was obtained. Accordingly, the present study suggests dual simultaneous dentistry applications of the synthesized AgNPs.
